# Increased Risk of Parkinson's Disease in Patients With Obstructive Sleep Apnea: A Population-Based, Propensity Score-Matched, Longitudinal Follow-Up Study

**DOI:** 10.1097/MD.0000000000002293

**Published:** 2016-01-15

**Authors:** Nai-Cheng Yeh, Kai-Jen Tien, Chun-Ming Yang, Jhi-Joung Wang, Shih-Feng Weng

**Affiliations:** From the Division of Endocrinology and Metabolism, Department of Internal Medicine, Chi Mei Medical Center (N-CY, K-JT); Department of Neurology (C-MY); Department of Medical Research, Chi Mei Medical Center (J-JW); Department of Healthcare Administration and Medical Informatics, Kaohsiung Medical University, Kaohsiung, Taiwan (S-FW); and Department of Senior Citizen Service Management, Chia Nan University of Pharmacy and Science, Tainan, Taiwan (K-JT).

## Abstract

Obstructive sleep apnea (OSA), characterized by repetitive episodes of apnea/hypopnea and hypoxia, is associated with systemic inflammation and induces metabolic, endocrine, and cardiovascular diseases. Inflammation might have an impact on neurodegenerative diseases. This study investigates the possible association between OSA and Parkinson's disease (PD). Random samples out of 1 million individuals were collected from Taiwan's National Health Insurance database. A total of 16,730 patients with newly diagnosed OSA from 2002 to 2008 were recruited and compared with a cohort of 16,730 patients without OSA matched for age, gender, and comorbidities using propensity scoring. All patients were tracked until a diagnosis of PD, death, or the end of 2011.

During the mean 5.6-year follow-up period, the incidence rates of PD were 2.30 per 1000 person-years in the OSA cohort and 1.71per 1000 person-years in the comparison group. The incidence rate ratio (IRR) for PD was greater in older patients (≧ 65 years) and male patients with OSA than the controls, respective IRRs being 1.34 and 1.47. After adjustment for the comorbidities, patients with OSA were 1.37 times more likely to have PD than patients without (95% CI = 1.12–1.68, *P* < 0.05). Subgroup analysis showed that older patients and patients with coronary artery disease, stroke, or chronic kidney disease had a higher risk for PD than their counter parts. Log-rank analysis revealed that patients with OSA had significantly higher cumulative incidence rates of PD than the comparison group (*P* = 0.0048). Patients with OSA are at an increased risk for subsequent PD, especially elderly male patients.

## INTRODUCTION

Obstructive sleep apnea (OSA) is a sleep disorder characterized by repeated upper airway collapse resulting in apnea/hypopnea and hypoxemia during sleep.^1^ Patients with OSA suffered from loud snoring, restless sleep, and excess daytime sleepiness. Approximately 20% of general population are affected by this disorder.^2,3^ OSA is thought to contribute to systemic disease and has been associated with many metabolic, endocrine, and especially cardiovascular diseases.^4–6^ The relationships between OSA and autoimmune disease and bone health including psoriasis, atopic dermatitis, and osteoporosis have also been investigated.^7–10^ Therefore, OSA is thought to be a complex disorder rather than a disease affecting the respiratory system only.

Parkinson's disease (PD) is a chronic, progressive degenerative disorder of the central nervous system carrying an incidence ranging from 8 to 18 per 100,000 person-years (PYs).^11^ It is thought that the motor symptoms of PD result from the death of dopamine-generating cells in the substantia nigra.^12^ The most obvious symptoms are movement-related and characterized by resting tremor, bradykinesia, rigidity, and postural instability. Later, cognitive problems may arise with dementia commonly occurring in the advanced stages of the disease. Several studies have linked oxidative stress with neurodegenerative diseases such as Alzheimer's disease, PD, other neural disorders, and aging.^13–15^

One study found a high percentage (71.8%) of snoring in patients with PD. Snoring has been associated with excessive daytime sleepiness.^16^ Some nonmotor symptoms observed in PD patients such as excessive daytime sleepiness, snoring, and cognitive impairment are similar with those in the sleep apnea syndrome although the mechanisms are different.^17^ Another study showed a higher incidence of snoring in patients with PD compared to healthy matched controls,^18^ and most of the snoring patients in that study fulfilled the criteria for obstructive sleep apnea (60%). Sleep disorders, especially OSA, have been further studied in PD patients.^19–21^ Cross-sectional studies have reported that sleep breathing disorders, with OSA seeming to be common in PD and often considered to be caused by PD. Cross-sectional design cannot confer the causality on observed associations. Without a well-designed longitudinal study, the causal relationship remains unclear between OSA and PD. To answer these questions, we used a population-based national insurance dataset to perform a longitudinal follow-up study to examine the causality between OSA and PD in Taiwan.

## METHODS

### Data Sources

This study used a national health insurance dataset released by the Taiwan National Health Research Institutes in 2011. NHI covers >98% of Taiwan's residents. Taiwan's National Health Insurance claims data is transferred into a research database by National Health Research Institutes. This database included details of all payments for outpatient visits, hospitalization, surgery, and medications of each patient. It also contains information on dose of drugs, special procedures intervention, and the dates of these treatments. One to 5 diagnoses codes were listed for every outpatient visits or hospitalization using the International Classification of Diseases, Ninth Revision. Because there was no recognizable individual information in the research data, informed consent was not necessary. The protocol of this study was reviewed and approved by the Institutional Review Board of Chi Mei Medical Center.

### Study Sample

This study enrolled 2 study groups: a newly onset OSA group and a matched non-OSA (comparison) group, both recruited within the year of 2002 to 2008. OSA was defined if the patient met the diagnostic criteria for that disease in at least 3 outpatient service claims with the codes for OSA (ICD-9-CM code 780.51, 780.53, 780.57, 327.23) at the department or division of Chest Medicine at any hospital or local medical clinic. Patients diagnosed as having OSA before 2002 were excluded. We also excluded patients who were diagnosed as having PD (ICD-9-CM code 332) before OSA. PD was defined if the patient met the relevant diagnostic criteria for that disease in at least 3 outpatient service claims with the codes for PD (ICD-9-CM code 332), inclusion criteria which has also been used in previous related studies.^22,23^ In this study, patients had to be diagnosed and followed up at the Division of Neurology at any hospital.

### Control Group

For each OSA case, 1 control without OSA was randomly selected in 2000 from the longitudinal Health Insurance Database 2000, which is a subset data of the National Health Insurance Research Database (NHIRD) and includes the 1996 to 2009 claims data of 1 million beneficiaries. To reduce the selection bias, the propensity score was used to match the comparison group by gender, age, and comorbidities. The benefit of propensity score is that it can bind confounding factors in an observational study. We first used a logistic regression model to analyze the dependent variables being the odds of diagnosis of OSA and the independent variables being baseline covariates (ie, age and gender) and the comorbidities we selected. Then, an SAS matching macro “%OneToManyMTCH” proposed in the *Proceedings of the 29th SAS Users Group International* was used to match the propensity score.^24^ Each control subject was selected at most once. Baseline comorbidities of these patients included diabetes mellitus (ICD-9-CM code 250), hypertension (ICD-9-CM code 401–405), coronary artery disease (CAD) (ICD-9-CM codes 410–414, A270, A279), stroke (ICD-9-CM codes 430–436), hyperlipidemia (ICD-9-CM codes 272.0–272.4), chronic kidney disease (ICD-9-CM codes 582, 583, 585, 586, 588), and gout (ICD-9-CM code 274) because these comorbid diseases may affect the risk of PD. These comorbid conditions were included if they were diagnosed in 3 or more ambulatory care claims or in an inpatient circumstance coded 1 year before the index date. The index date for OSA was the date of first administration and the index date for the comparison group was established by matching the index date of OSA patients. Follow-up time in person-years (PYs) was analyzed for each person until PD was diagnosed, death, or the end of 2011. Our outcome of interest, PD, had to be diagnosed and followed up at Division of Neurology of any hospital.

### Statistical Analyses

Pearson χ2 tests were used to compare the difference between 2 groups in gender, age, and comorbidities we selected. The incidence rate was computed as the number of PD patients during the follow-up divided by per 1000 person-years for each group by the gender and the age. We also used Cox proportional hazard regression to examine the risk of PD between the OSA and controls. Kaplan–Meier analysis was used to estimate the cumulative incidence rates of PD between 2 cohorts. The log-rank test was used to compute the differences between the survival curves. A 2-sided value of *P* < 0.05 was considered significant. All statistical calculations were performed using the SAS 9.4 statistical package (SAS Institute, Inc).

## RESULTS

Table 1 showed the baseline characteristics and comorbidities of the study groups. We followed up 16,730 OSA patients and 16,730 propensity-score matched non-OSA cohorts for 5.6 years. Almost 51% (50.9%) of the patients were 0 to 50 years old, 32.4% were 50 to 64 years old, and 16.5% were 65 years or older. Eighty-one percent were men. Patients with OSA tended to have comorbidities such as diabetes (10.3%), hypertension (32.3%), CAD (11.1%), stroke (4.6%), hyperlipidemia (13.2%), chronic kidney disease (1.7%), and gout (6.7%). Because patients and controls were matched for comorbidities, the percentages of the comorbidities were the same in the OSA and the control groups.

**TABLE 1:**
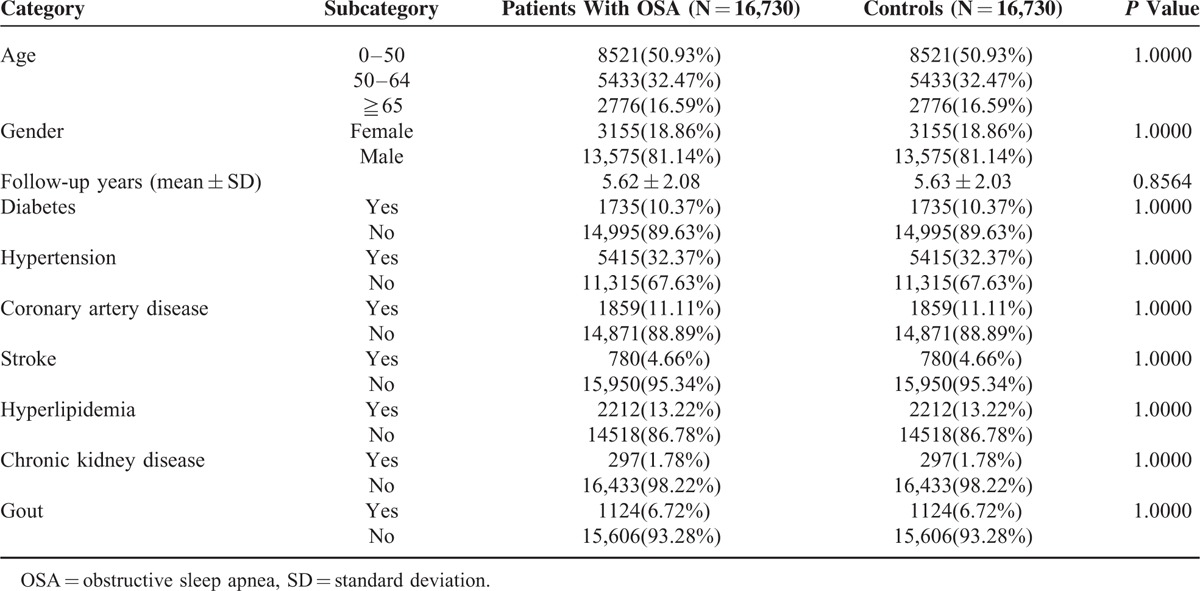
Demographic Characteristics and Comorbid Medical Disorders for OSA Patients and Comparison Group in Taiwan

As is seen in Table 2, the OSA group had a significantly higher incidence of PD than the control group. A total of 217 of the 16,730 OSA patients were diagnosed as having PD during the follow-up (2.30 per 1000 PYs). A total of 161 of 16,730 controls developed PD during the follow-up period (1.71 per 1000 PYs), making an incidence rate ratio (IRR) of 1.34 (95% confidence interval [CI] 1.09–1.64; *P* = 0.0048). After matching age groups, the difference remained significant in the elderly (>65 years old) (IRR 1.34, 95% CI 1.04–1.73, *P* = 0.023). Men with OSA had a significantly higher risk for PD than those without OSA (IRR 1.49, 95% CI 1.16–1.86, *P* = 0.0016). Women with OSA did not have a higher risk for PD than those without (IRR 1.07, 95% CI 0.71–1.59, *P* = 0.7574).

**TABLE 2:**
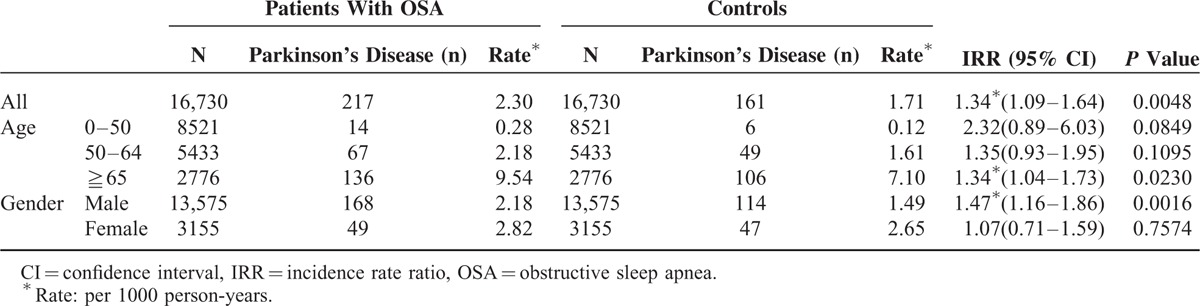
Risk of Parkinson's Disease for Obstructive Sleep Apnea (OSA) Patients and Controls

**FIGURE 1:**
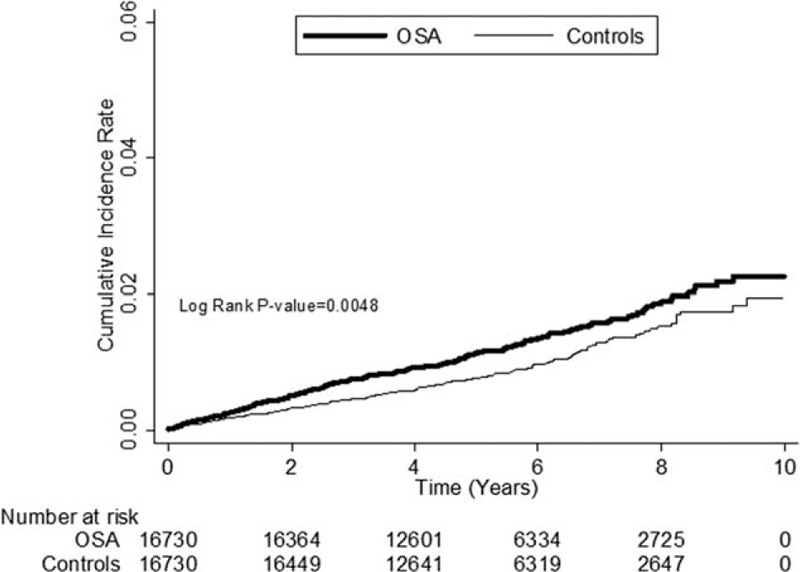
The cumulative incidence rate for Parkinson's disease for patients with obstructive sleep apnea (OSA) and controls (log-rank *P* value = 0.0048). OSA = obstructive sleep apnea.

In Table 3, the crude hazard ratio (HR) for PD in patients with OSA was 1.34, compared with those without (95% CI 1.09–1.64, *P* < 0.05). After adjusting for age, gender, diabetes, hypertension, CAD, stroke, hyperlipidemia, chronic kidney disease, and gout, we found patients with OSA to be 1.37 times more likely to develop PD during the follow-up period (adjusted HR 1.37, 95% CI 1.12–1.68, *P* < 0.05). No significant gender difference in the development of PD was found after adjustment. Older patients had a higher risk for PD than their younger counterparts (aged < 50 years). The adjusted HR was 8.32 (95% CI 5.15–13.48, *P* < 0.05) in those aged 50 to 64 years and 30.60 in those aged 65 years or older (95% CI 19.00–49.28, *P* < 0.05) compared with subjects aged 0 to 50 years. Other significant factors included coronary artery disease (adjusted HR 1.32, 95% CI 1.05–1.67, *P* < 0.05), stroke (adjusted HR 2.12, 95% CI 1.63–2.77, *P* < 0.05), and chronic kidney disease (adjusted HR 2.27, 95% CI 1.51–3.42, *P* < 0.05). Kaplan–Meier analysis revealed that patients with OSA had a higher incidence of PD than the control cohort (log-rank test *P* < 0.0048) (Figure 1).

**TABLE 3:**
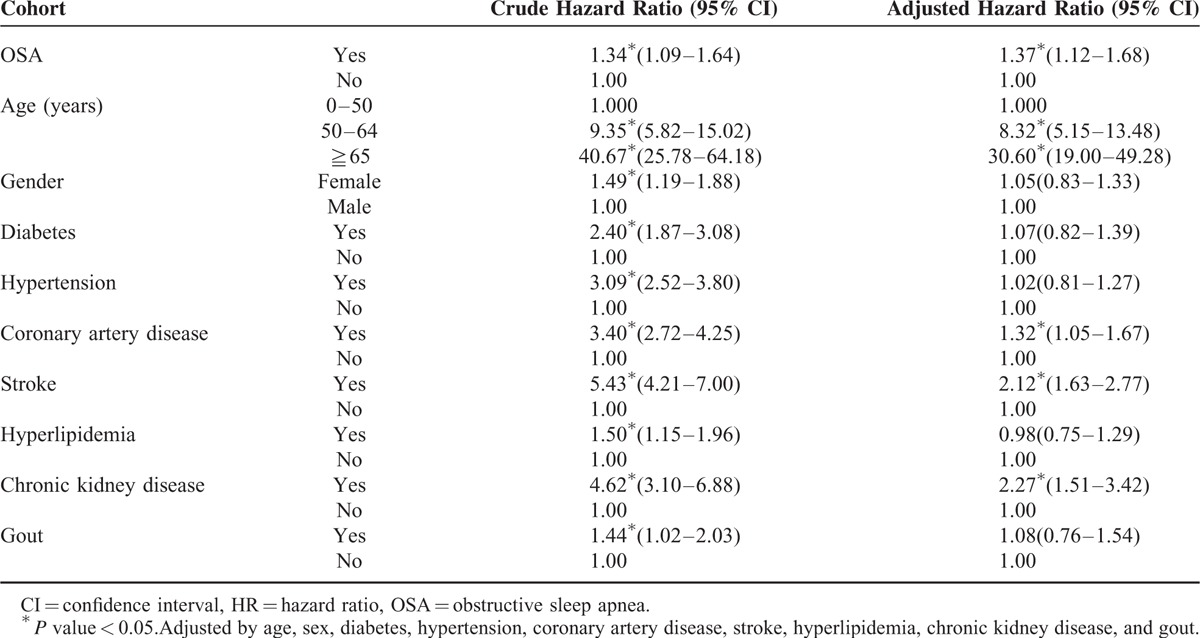
Crude and Adjusted Hazard Ratios (HR) for the Development of Parkinson's Disease Among the Study Cohort

## DISCUSSION

OSA has been associated with many diseases causing systemic inflammation and vascular atherosclerosis.^4–7,9,25^ Recent studies show that incidence of OSA is similar between PD patients and healthy controls^21,26,27^ and in patients with PD the effect of OSA on sleepiness may be small^17^ and sympathetic response to OSA is blunted.^28^ However, because the relationship between PD severity and OSA is still controversial,^29^ we studied a population-based cohort to evaluate the relationship between OSA and PD. The present study represents the largest population-based cohort study to evaluate the association of OSA and PD in a 5.6-year follow-up investigation of an Asian population. This study found that the incidence of PD to be 1.37 times higher in patients with OSA than the comparison cohort matched by propensity-score after adjusting for age, gender, and medical comorbidities. Subgroup analysis found the hazard of developing PD to be greater in older patients and in those with CAD, stroke, and chronic kidney disease.

OSA induces brain hypoperfusion, increasing oxidative stress, leading to pathogenic effects on endothelial dysfunction.^30,31^ Increased oxidative stress has been independently associated with OSA severity.^32^ Oxidative stress also plays an important role in neurodegenerative diseases, including Alzheimer's disease, PD, aging, and many other neural disorders.^13–15^ Oxidative stress and inflammation, 2 mechanisms linked to OSA, have also been reported to be involved in the pathophysiology of PD.^33,34^

PD is associated with loss of dopamine-secreting neurons within the substantia nigra. The presence of Lewy bodies is the major pathological feature of inside nerve cells in PD. Early in the course of disease, dopamine deficiency in addition to serotoninergic, cholinergic, and noradrenergic systems is the predominant neurochemical abnormality.^35,36^ There is evidence showing astrocytic dopamine D2 receptor (DRD2) modulates innate immunity through αB-crystallin (CRYAB), which is known to suppress neuroinflammation. Knockout mice lacking DRD2 have been found to have remarkable inflammatory responses in multiple central nervous system regions as well as increased neurotoxicity.^37^ C-reactive protein (CRP) which is a biomarker of inflammation is associated with the risk of death and predicted prognosis of patients with PD, also suggesting the possibility that chronic inflammation is associated with a neurodegenerative process in PD.^38^ Inflammation may play an important role of PD. The intermittent hypoxia associated with sleep apnea may also increase the loss of dopaminergic neurons, thereby worsening motor disability. Dopamine pathways are vulnerable to ischemic-anoxic insult.^39^ By now, the underlying mechanism linking OSA and PD is not well validated. The oxidative stress, systemic inflammation, and the procoagulant and thrombotic activity discovered in OSA may be the key.^40^

The men with OSA in our study were at a significantly higher risk for PD. Endogenous and exogenous estrogen exposures are proposed to be responsible for sex differences in epidemiologic researches. Estrogen may have neuroprotective effects by reducing the dopaminergic neuron depletion caused by neurotoxins in the PD animal study.^41^ A recent study showed a greater susceptibility to PD in men with lower sex hormone and higher serum prolactin level.^42^ Thus, it may be possible that sex hormone plays a role in inducing or worsening PD, though studies of testosterone treatment in men suffering from PD show equivocal results.^43,44^

Increased risk of PD with age has also been found.^45^ In our study, those aged 50 to 64 years had 8.3 times the risk and aged 65 years or older had the 30.6 times the risk of PD compared with subjects aged 40 to 49 years. Patients with CAD, stroke, and chronic kidney disease also had a higher risk of PD, possibly suggesting contributions of vascular disease and atherosclerosis in the development of PD.^46^ Elevated plasma levels of homocysteine, which can be found in cardiovascular disorders and atherosclerosis, have also been observed in PD.^47^

Sheu et al^48^ also has reported OSA to be a risk factor for subsequent PD. Among females, the adjusted hazard ratio of PD was 3.54 (95% CI = 1.50–8.34) for patients with OSA compared to patients without OSA. Their reported risk of PD between genders is inconsistent with the results of the present study. As we know, there are more men than women suffering from severe sleep-disordered breathing (apnea–hypopnea Index (AHI) ≥15) recorded by the Wisconsin cohort study^49^ and the updated population prevalence study.^50^ There is a strong association between OSA severity and elevated erythrocyte sedimentation rate (ESR), a representative marker for inflammation.^51^ It is more reasonable that men have higher risk of PD as we assume inflammation plays an important role of PD. The inconsistency might also be explained by our use of propensity scores to adjust the comorbidities. This can greatly attenuate the influence of the comorbidities on PD outcome. In addition, the criteria use in this study for a diagnosis of PD diagnosis is more precise than the definitions used by previous similar studies.

However, this study also has several limitations. One limitation was that the identification of OSA and PD was based on diagnostic codes listed in the database. Therefore, some coding errors might have occurred. However, the NHI Research Database has been found to have acceptable validity for epidemiologic investigations.^52^ Coding of OSA from Taiwan's National Health Insurance database had been validated in previous related studies.^53^ To maximize case ascertainment, we used the criteria: at least 3 outpatient service claims with the codes of OSA (ICD-9-CM code 78051, 78053, 78057, 32723) at department or division of Chest Medicine of any hospital. This would help improve the validity of diagnosis. The same method was used for PD (ICD-9-CM code 332) diagnosis, which had also been used in previous related studies.^22,23^ In our study, patients had to be diagnosed and followed up at the Division of Neurology at any hospital. This added criterion may have improved the accuracy of the coding. Another limitation may be that individual information, including the severity of diseases, environmental factors, family history, herbal medicine, diet, smoking, and alcohol use, were not accessible. The propensity-score matching was used to reduce the selection bias in our study. However, only apparent factors can be used in the analyses and some unknown or unobservable variables may still confound the results.

In conclusion, our study found that patients with OSA had a higher risk of developing PD, especially in elderly people and men. Physicians treating patients with OSA should keep this association in mind, as PD interferes with a person's physical movement and the quality of life in later years. Further study is needed to evaluate the possible underlying mechanisms underlying OSA's effect on PD.
